# Compliance with hand hygiene practices among nursing staff in secondary healthcare hospitals in Kuwait

**DOI:** 10.1186/s12913-022-08706-8

**Published:** 2022-11-08

**Authors:** Sarah Al-Anazi, Nora Al-Dhefeery, Rawan Al-Hjaili, Awsaf Al-Duwaihees, Ahad Al-Mutairi, Reem Al-Saeedi, Retaj Al-Dhaen, Sara Al-Rabiah, Reem Sharaf-Alddin

**Affiliations:** 1 Departments of a Community Medicine and Behavioral Science, Faculty of Medicine, Kuwait University, Jabriya, Kuwait; 2 CONARD, East Virginia Medical School, VA Norfolk, USA

**Keywords:** Compliance, Hand hygiene, Nurse, My 5 moments for hand hygiene

## Abstract

**Background:**

Hand hygiene (HH) among healthcare workers, especially nurses, is the main preventive measure to control healthcare associated infections but compliance with hand hygiene (CwHH) remains low in various settings including Kuwait. This study aimed to assess the knowledge of, attitudes towards, and CwHH among nursing staff in secondary care hospitals in Kuwait.

**Methods:**

A cross-sectional study was conducted on nursing staff in all six secondary care hospitals in Kuwait. Data on knowledge of, attitudes towards, and self-reported CwHH were collected through a self-administered questionnaire that was developed based on WHO’s questionnaire, while the data on actual compliance were objectively collected through direct observation of nurses during routine care by two independent observers using WHO’s observation form.

**Results:**

Of 829 nurses approached, 765 (92.2%) responded and participated. Of all participants, 524 (68.5%) were able to list “My Five Moments for Hand Hygiene” fully and appropriately. However, several misconceptions (e.g. air circulation in hospital is the main route of infection) about HH were found among the nurses. CwHH was (25.0%) by direct observation while self-reported compliance was (69.5%) each varied significantly (*p* < 0.001) between different hospitals. Female nurses compared to male nurses and non-Arab compared to Arab nationalities were more likely to report CwHH in multivariable analysis. Several items on knowledge of and attitudes towards HH were also associated with self-reported CwHH.

**Conclusion:**

Observed CwHH among nursing staff in secondary care hospitals in Kuwait was low, which highlights the need to make more efforts to improve HH practices. Interventions that have been used elsewhere and found to be effective may be tested in Kuwait. Despite the good overall knowledge on HH among nurses, there are several misconceptions that need to be corrected.

## Introduction

World Health Organization (WHO) has defined healthcare associated infections (HCAIs) as “that affect patients during the process of care in hospitals or other healthcare facilities, which were not present or incubating at the time of admission [1, 2]”. HCAIs are a major public health problem, which lead to prolonged hospital stays [3, 4], high mortality [5], long-term disability, and excess healthcare costs [6]. WHO estimated that about 7% of hospitalized patients in developed countries suffer from HCAI [7], while in developing countries the pooled prevalence of HCAI is 15% [8]. In European countries, the burden of HCAIs was estimated to be 170 Disability-adjusted Life Years (DALYs) per 100,000 population with more impact among infants and older individuals [9].

Hand Hygiene (HH) among healthcare workers, especially nurses, is the main preventive intervention to control HCAIs [10–14]. Although the techniques involved in HH are simple, compliance with hand hygiene (CwHH) recommendations is poor worldwide [15, 16], particularly in Intensive Care Units (ICUs), where CwHH ranges from 64% in high income settings to as low as 9% in low income settings [17, 18]. In a systematic review of studies on CwHH in hospitals in industrialized countries, the CwHH was estimated to be 40% which was even lower in ICUs [19].

In an attempt to reduce the burden of HCAIs, the WHO introduced “My Five Moments for Hand Hygiene” in 2009 [20], which defined the key moments of HH as before touching the patient, before clean/aseptic procedure, after exposure to body fluids, after touching the patient, and after touching the patient’s surroundings. This approach is now widely used to assess CwHH in research studies as well as in routine audit of HH in healthcare facilities.

In Kuwait, although HCAIs are around (10.6%) [21], only one single study has attempted to explore the knowledge of HH and CwHH among nursing staff [22]. The study was conducted more than a decade ago and before the WHO’s guidelines on HH become commonly used. The authors reported poor CwHH (33.4%) among nursing staff in secondary healthcare hospitals despite the high level of awareness of HH [22]. Therefore, in this study we aimed to estimate CwHH in hospitals in Kuwait and assess the knowledge and attitudes towards HH among nursing staff in secondary healthcare hospitals.

## Methods

### Study sites and study population

In Kuwait, public health services comprise primary healthcare centers, secondary healthcare hospitals, and tertiary hospitals and centers. Private healthcare services are also available in terms of private clinics, private hospitals and private specialized tertiary centers. In public healthcare hospitals, nursing staff represent almost half of the employees in these hospitals accounting for 22,000 nurses. Patients’ rooms in hospitals are either large rooms (four hospital beds in each room) in which there is one hand wash basin with one antibacterial hand soap dispenser and one hand sanitizer in addition to hand sanitizer besides each hospital bed, or small private rooms (one hospital bed in each room) in which there is one hand wash basin with one antibacterial hand soap dispenser and one hand sanitizer in addition to one hand sanitizer besides hospital bed. In all hospitals, "My Five Moments for Hand Hygiene" posters are displayed in the notice boards in each ward to raise the awareness about the importance of HH practices.

### Sampling and study design

**Fig. 1:**
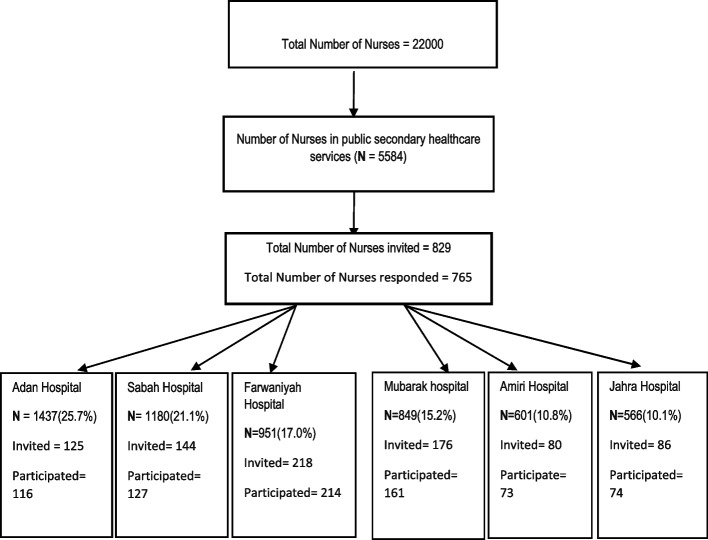
The total number of nurses, number of nurses selected, and number of nurses participated

This is a cross-sectional study that was conducted before COVID-19 pandemic in March–April 2019 among nurses in all secondary healthcare hospitals who are working in pediatrics, surgery, emergency, and medicine wards. The number of nurses selected from each was proportional to the total number of nurses in that hospital compared to the total number of nurses in all the six hospitals. The total number of nurses in each hospital as well as the number of nurses selected and participated is shown in Fig. 1.

### Data collection

Data collection was done by two methods; direct observation by the researchers, and self-administered questionnaire completed by the study participants (nurses). Before the direct observation, the study was explained to the head nurse, who was requested not to inform the ward nurses about the study.

#### Direct observation

Data on actual compliance was collected by direct observation using the standard WHO’s observation form of “My Five Moments for Hand Hygiene” which defines the key moments when healthcare workers should perform HH (before touching the patient, before clean/aseptic procedure, after exposure to body fluids, after touching the patient, and after touching the patient’s surroundings) [23]. This method was used in order to standardize the procedure of assessing CwHH and minimizing the differences in understanding the indications for HH during delivery of healthcare among healthcare workers. Direct observations were conducted in the six secondary healthcare hospitals over seven days and in different shifts (i.e. morning, evening and night shifts). Each observation session lasted for 20 min in which the observer stood close to the point of care and recorded the HH moments which refer to the number of times at which the HH should be performed. In sequence with that, the observer recorded the HH actions which refers to the number of times in which nurse practiced HH. To assess the inter-observer variability, the observation was conducted in each session by two independent observers at the same time and the two observers independently recorded number of times when HH should be performed (HH moments) and the number of times when HH actions occurred. A HH action was defined as either rubbing hands with an alcohol-based hand rub or hand washing with soap. Compliance was calculated as:

$$\mathrm{Compliance}\%\;=\frac{number\mathit\;of\mathit\;hand\mathit\;hygiene\mathit\;actions}{number\mathit\;of\mathit\;hand\mathit\;hygiene\mathit\;moments}\;\times100$$

Hawthorne effects occurs when people behave better than usual if they are aware that they are being observed (i.e. nurses may modify their hand hygiene practices in response to their awareness of being observed) [24]. To minimize "Hawthorne effect", only head nurse was aware of the observation, and he/she was asked not to inform the nursing staff about the study. Also, observers were senior medical students whom their presence at hospital wards is part of normal daily routine, and they did not reveal any information about the nature of the study.

#### Self-administered questionnaire

After collecting data by direct observation, data on knowledge of, attitude towards, and self-reported CwHH were collected by self-administered questionnaire. The questionnaire included questions on socio-demographic factors, professional status, knowledge (using nine questions from the WHO’s hand hygiene knowledge questionnaire) [25], and attitudes (using 11 statements with “agree” or “disagree” answers for each statement). Self-reported CwHH among nurses was investigated using four patient care scenarios (before insertion of IV cannula, before changing bed sheets, before measuring patient’s temperature, and before measuring patient’s blood pressure) during which HH is absolutely required based on the WHO’s Hand Hygiene Technical Reference Manual [23]. In each scenario, nurses were asked about what they usually do in terms of HH (HH action: hand wash, hand rub, gloves, or none) and how frequent is their action (always, often, sometimes, or never). Participants who reported that “they always perform hand wash and/or hand rub” in all four scenarios were considered compliant, while the rest were considered non-compliant.

### Statistical methods

Data were analyzed using IBM Statistical Package for Social Sciences (SPSS) Statistics version 25. Age was summarized by mean (SD) after checking for normality. Categorical variables were summarized by frequencies and percentages. The Chi-squared test was used to assess the differences in self-reported CwHH (Compliant vs. Non-compliant) between different hospitals. While binary logistic regression was used to calculate the crude and adjusted odds ratio for the association between presumed factors and self-reported CwHH.

## Results

Of 829 nurses invited to the study, 765 (92.2%) responded and participated. Table 1 shows the socio-demographic characteristics of the study participants. The mean (SD) age was 36.8 (7.4) years, of whom 586 (76.6%) were females. More than two thirds of the nurses 582 (76.2%) were Indians, while only 19 (2.5%) nurses were Kuwaitis.

**Table 1:** Socio-demographic characteristics of 765 nursing staff in six secondary care hospitals in Kuwait

**Variable**	
**Age**	**Mean (SD)**
	36.82 (7.4)
**Gender**	**n (%)**
**Female**	586 (76.6)
**Male**	179 (23.4)
**Nationality**^**a**^	**n (%)**
**Kuwaiti**	19 (2.5)
**Filipino**	62 (8.1)
**Indian**	582 (76.2)
**Egyptian**	19 (2.5)
**Other Arab**	63 (8.2)
**Other Non-Arab**	19 (2.5)
**Salary**	**n (%)**
**200-300KD**	23 (3.0)
**301-400KD**	49 (6.4)
**401-500KD**	49 (6.4)
**More than 500KD**	627 (82.0)
**Not willing to report**	17(2.2)
**Marital status**^**b**^	**n (%)**
**Married**	675 (88.4)
**Non married**	79 (10.3)
**Divorced/widow**	10 (1.3)

^a^Missing for one participant

^b^Missing for one participant

### Knowledge of and attitude towards hand hygiene

The majority (98.6%) of nursing staff have heard about “My Five Moments for Hand Hygiene”, of whom (93.0%) reported they are able to list them. Of all participants, 524 (68.5%) were able to list “My Five Moments for Hand Hygiene” fully and appropriately. Only (53.6%) of the participants reported that they have received formal training on HH during the last two years. Table 2 shows the knowledge of nursing staff on hand hygiene. About two thirds of the nurses 563 (73.6%) recognized that the main route of transmission of harmful germs in healthcare facilities is the contaminated hands of healthcare workers, while a very small number 53 (6.9%) nurses thought air circulation in healthcare facilities is the main route of transmission. Only a few participants 29 (3.8%) said they do not know the main route of transmission of germs in healthcare facilities. Almost all nurses (96.5%) recognized that HH before touching a patient will help prevent transmission of germs to that patient. However, only 145 (19.0%) nurses knew that the most frequent source of germs responsible for healthcare associated infections is present on or within the patient.

**Table 2:** Knowledge about, and Attitude toward hand hygiene among 765 nursing staff in six secondary health care hospitals in Kuwait

**Variable**	**n**	**(%)**
**a. Knowledge about hand hygiene**		
**Question (correct answer)**		
**Which of the following is the main route of cross-transmission of potentially harmful germs between patients in a health care facility? n (%) (**Health-care workers’ hands when not clean)	563	(73.6)
**What is the most frequent source of germs responsible for healthcare associated infections? n (%)** (germs already present on or within the patient)	145	(19.0)
**Which of the following hand hygiene actions prevents transmission of germs to the patient?**		
Before touching a patient, **n (%)** (yes)^a^	737	(96.5)
Immediately after risk of body fluid exposure, **n (%)** (yes)^a^	576	(75.4)
After exposure to immediate surroundings of a patient, **n (%)** (no)^b^	121	(15.9)
Immediately before clean/aseptic procedure, **n (%)** (yes)^a^	677	(88.6)
**Which of the following hand hygiene actions prevents transmission of germs to the health-care worker?**		
After touching the patient, **n (%)** (yes)^a^	721	(94.4)
Immediately after risk of body fluids exposure, **n (%)** (yes)	713	(93.2)
After exposure to immediate surroundings of a patient, **n (%)** (yes)^a^	686	(89.8)
Immediately before clean/aseptic procedure, **n (%)** (no)^a^	152	(19.9)
**Which of the following statements on alcohol-based hand rub and hand washing with soap and water are true?**		
Hand rubbing is more rapid for hand cleansing than hand washing, **n (%)** (true)^b^	120	(15.7)
Hand rubbing causes skin dryness more than hand washing, **n (%)** (false)^a^	391	(51.2)
Hand rubbing is more effective against germs than hand washing, **n (%)** (false)^a^	188	(24.6)
Hand washing and hand rubbing are recommended to be performed in sequence, **n (%)** (false)	511	(66.8)
**Which type of hand hygiene method is required in the following situations?**		
Before palpation of the abdomen, **n (%)** (rubbing)	609	(79.6)
Before giving an injection, **n (%)** (rubbing)	312	(40.8)
After emptying a bedpan, **n (%)** (washing)	711	(92.9)
After removing examination gloves, **n (%)** (rubbing/washing)	749	(98.0)
After making a patient’s bed, **n (%)** (rubbing)	322	(42.1)
After visible exposure to blood, **n (%)** (washing)	710	(92.8)
**Which of the following should be avoided, as associated with increased likelihood of colonization of hands with harmful germs?**		
Wearing jewelry, **n (%)** (yes)^a^	712	(93.2)
Damaged skin, **n (%)** (yes)	677	(88.5)
Artificial nails, **n (%)** (yes)^a^	716	(93.7)
Regular use of hand cream, **n (%)** (no)^a^	426	(55.8)
**Attitude toward hand hygiene**		
Statement n (%)	Agree	Disagree
Do you agree or disagree with the following statements?		
I adhere to correct hand hygiene practices at all times	735 (96.1)	30 (3.9)
I have sufficient knowledge about hand hygiene	726 (94.9)	39 (5.1)
Sometimes I have more important things to do than hand hygiene	192 (25.1)	573 (74.9)
Emergencies and other priorities make hand hygiene more difficult sometimes	456 (59.6)	309 (40.4)
Wearing gloves reduces the need for hand washing^a^	194 (25.4)	570 (74.6)
I feel guilty if I omit hand washing	650 (85.0)	115 (15.0)
Adhering to hand washing practices is easy in the current setup^b^	664 (87.0)	99 (13.0)
I feel frustrated when others omit hand hygiene	624 (81.6)	141 (18.4)
Hand hygiene is an essential part of my role	727 (95.0)	38 (5.0)
Sometimes I miss out hand hygiene simply because I forget it	162 (21.2)	603 (78.8)
The frequency of hand hygiene required makes it difficult for me to carry it out as often as necessary^b^	200 (26.2)	563 (73.8)

^a^Missing for one participant

^b^Missing for two participants

About two thirds 573 (74.9%) of the nurses disagreed with the statement "sometimes I have more important things to do than hand hygiene”, and almost all of them 727 (95%) felt that HH is an essential part of their role (Table 2). The majority 664 (87.0%) of the nurses agreed that adhering to hand washing practices is easy in the current setup.

### Directly observed compliance with hand hygiene

Table 3 shows the CwHH by direct observation over a one-week period, the observation was conducted in 194 sessions, each lasted 20 min (more than 64 h in total). The total number of HH moments reported were 2307 by observer 1 and 2315 by observer 2 (difference in counting moments 0.35%). Of those, only 570 and 580 HH actions were observed by observer 1 and 2 respectively (difference in counting HH actions 1.7%). The overall compliance by direct observation was around (25.0%) and varied significantly between hospitals (*p* < 0.001).

**Table 3:** The observed compliance with hand hygiene among nursing staff in secondary health care hospitals

**Hospital**	**Number of sessions**	**Number of moments**		**Number of actions**		**Compliance**	
		**Observer 1**	**Observer 2**	**Observer 1**	**Observer 2**	**Observer 1**	**Observer 2**
**Amiri**	24	197	196	25	22	12.7%	11.2%
**Adan**	41	369	366	32	36	8.7%	9.8%
**Mubarak**	36	355	359	85	87	23.9%	24.2%
**Jahra**	20	370	373	88	90	23.7%	24.1%
**Farwaniyah**	37	701	707	195	201	27.8%	28.4%
**Sabah**	36	315	314	145	144	46.0%	45.8%
**Total**	194	2307	2315	570	580	24.7%	25.0%

### Self-reported compliance with hand hygiene

**Fig. 2:**
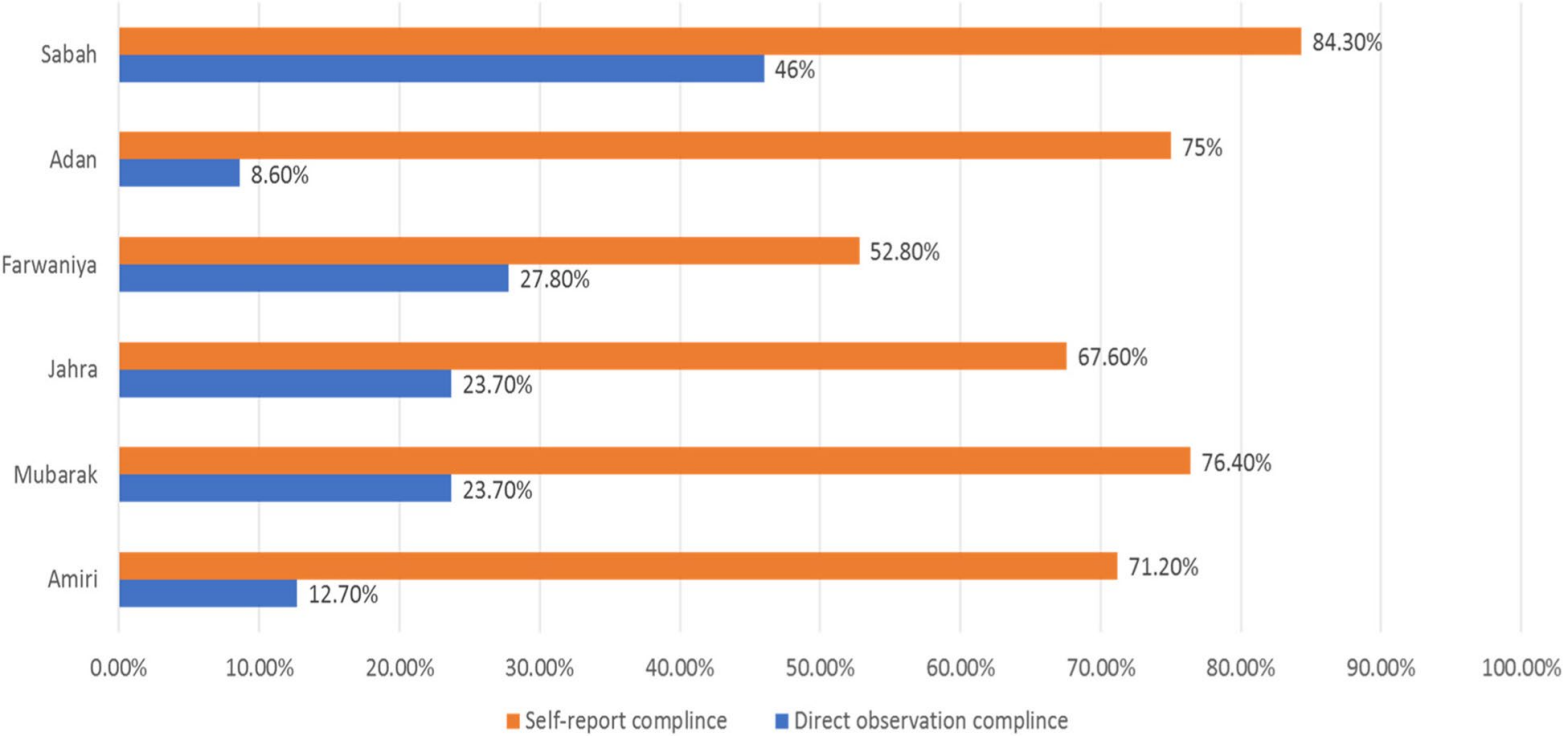
Direct observation and self-report compliance in secondary health care hospitals

Self-reported CwHH was (69.5%), which varied significantly between different hospitals (Fig. 2) (*p* < 0.001). This difference remained evident even after stratification by the type of unit. Factors that were significantly associated with self-reported compliance in univariable analysis were gender (*p* = 0.021), nationality (*p* = 0.004), marital status (*p* = 0.051), hospital (*p* < 0.001), unit (*p* < 0.001), number of morning shifts per week (*p* = 0.051), and number of night shifts per week (*p *= 0.004). Additionally, there are several questions that measure the knowledge of, attitudes towards HH that were significantly associated with self-reported CwHH in univariable analysis.

Table 4 shows the factors that were significantly associated with self-reported CwHH in both univariable and multivariable analysis. Females were more likely to self-reported CwHH than males, (adjusted OR: 1.63, [95%CI: 1.07–2.48]; *p* = 0.023). Similarly, nurses of non-Arab nationalities (including Indians) were more likely to report CwHH than Arabs (*p* = 0.014). CwHH was significantly different by unit, with nurses working in surgical (adjusted OR: 3.71, [95%CI: 2.04 – 6.75]), medical (adjusted OR: 2.74, [95%CI: 1.55 – 4.84]) and pediatric (adjusted OR: 3.36, [95%CI: 1.81 – 6.23]) wards were more likely to report CwHH compared to nurses working in emergency (*p* < 0.001). Although several items that measure knowledge of HH were significantly associated with self-reported CwHH in univariable analysis, only a few found to be significantly associated with CwHH in multivariable analysis (see Table 4). The correct answer for the question on whether HH immediately before clean/aseptic procedure prevents transmission of germs to the patient was significantly associated with self-reported CwHH in both univariable and multivariable analysis (adjusted OR: 2.12 [95%CI: 1.25–3.59]; *p* = 0.005). Likewise, the correct answer for the question on whether HH after exposure to immediate surroundings of a patient prevents transmission of germs to the healthcare worker was also associated with self-reported CwHH before and after adjusting for other factors (adjusted OR: 2.28, [95%CI: 1.27–4.07]; *p* = 0.005). Only two statements on attitudes were significantly associated with self-reported CwHH in multivariable analysis. Nurses who disagreed with the statement that they adhere to correct hand hygiene practices at all times were less likely to self-report CwHH (adjusted OR: 0.261, [95%CI: 0.12–0.59]; *p* = 0.001). In contrast, nurses who disagreed with the statement that wearing gloves reduces the need for hand washing were more likely to self-report CwHH (adjusted OR:1.61, [95%CI: 1.09–2.38]; *p* = 0.06).

**Table 4:** Association between self-reported compliance with hand hygiene and several predictors in univariable and multivariable analysis

**Variables**	**N**	**Compliance n (%)**	**Crude**			**Adjusted**		
			**OR**	**(95%CI)**	***p*-value**	**OR**	**(95%CI)**	***P*-value**
**Hospital**								
Amiri	73	52 (71.2)	1.00	(Reference)	< 0.001	1.00	(Reference)	< 0.001
Mubarak	161	123 (76.4)	1.30	(0.70–2.43)		1.37	(0.68–2.73)	
Jahra	74	50 (67.6)	0.84	(0.41–1.69)		0.93	(0.42–2.08)	
Farwaniya	214	113 (52.8)	0.45	(0.25–0.80)		0.59	(0.31–1.12)	
Adan	116	87 (75.0)	1.21	(0.62–2.34)		1.12	(0.54–2.31)	
Sabah	127	107 (84.3)	2.16	(1.07–4.33)		2.33	(1.05–5.13)	
**Unit**								
**Emergency**	205	152 (74.1)	1.00	(Reference)	< 0.001	1.00	(Reference)	< 0.001
**Medical ward**	297	201 (67.7)	2.62	(1.57–4.39)		2.74	(1.55–4.84)	
**Pediatrics**	171	131 (76.7)	1.91	(1.19–3.08)		3.36	(1.81–6.23)	
**Surgical ward**	92	48 (52.2)	3.00	(1.74–5.15)		3.71	(2.04–6.75)	
**Gender**								
**Male**	179	112 (62.6)	1.00	(Reference)	0.021	1.00	(Reference)	0.023
**Female**	586	420 (71.7)	1.51	(1.06–2.15)		1.63	(1.07–2.48)	
**Nationality**								
**Arabs**	101	56 (55.4)	1.00	(Reference)	0.004	1.00	(Reference)	0.014
**Indian**	582	417 (71.6)	2.03	(1.31–3.12)		2.05	(1.24–3.38)	
**Other non-Arab**	81	59 (72.8)	2.25	(1.15–4.03)		2.24	(1.08–4.62)	
**Hand Hygiene prevents transmission of germs to the patient (immediately before clean\aseptic procedure)**								
**Incorrect**	87	39 (44.8)	1.00	(Reference)	< 0.001	1.00	(Reference)	0.005
**Correct (Yes)**	677	493 (72.8)	3.29	(2.09–5.19)		2.12	(1.25–3.59)	
**Hand Hygiene prevents transmission of germs to the health-care worker (after exposure to immediate surroundings of a patient)**								
**Incorrect**	78	34 (43.6)	1.00	(Reference)	< 0.001	1.00	(Reference)	0.005
**Correct (Yes)**	686	498 (72.6)	3.42	(2.12–5.52)		2.28	(1.27–4.07)	
**I adhere to correct hand hygiene practices at all times**								
**Agree**	735	521 (70.9)	1.00	(Reference)	< 0.001	1.00	(Reference)	0.001
**Disagree**	30	11 (36.7)	0.238	(0.11–0.50)		0.26	(0.11–0.59)	
**Wearing gloves reduce the need for hand washing**								
**Agree**	194	111 (57.2)	1.00	(Reference)	< 0.001	1.00	(Reference)	0.016
**Disagree**	570	420 (73.6)	2.09	(1.49–2.94)		1.61	(1.09–2.38)	

## Discussion

This cross-sectional study aimed to estimate the knowledge of, attitude towards, and self-reported CwHH among nursing staff in secondary healthcare hospitals in Kuwait. We also collected data on CwHH by direct observation according to the WHO’s hand hygiene guidelines. We found that nursing staff have good knowledge on some aspects of HH but only around a quarter of nurses were compliant with hand hygiene guidelines by direct observation.

In our study, the majority (98.6%) of nurses have heard about “My Five Moments for Hand Hygiene”, but only (68.5%) were able to list them fully and appropriately. However, this is far better than that reported from the US (Maryland), where only (29.0%) of the healthcare personnel were familiar with “My Five Moments for Hand Hygiene” and only (6.0%) were able to recall them [26]. Our findings can be explained by the fact that hospitals were seeking accreditation during the study period hence put emphasis on HH practices to reduce HCAIs (more than half of the nurses received formal training during the past two years). Furthermore, (73.6%) of nursing staff recognized contaminated healthcare workers’ hands as the main route of transmission of germs to patients in healthcare facilities, which is similar to another study in Saudi Arabia, where (77.8%) of nursing staff identified the contaminated hands of healthcare workers as the main route of transmission [27]. However, in our setting around (6.9%) thought that air circulation in healthcare facilities is the main route of infection. Data collection in this study was before COVID-19 pandemic, and despite that the transmission of SARS-CoV-2 through air circulation is possible, it is not the main route of transmission. We found only (19.0%) of nurses were aware that the most frequent source of germs responsible for healthcare associated infections is present on or within the patient, which is low compared to a study in Biratnagar, in Nepal (30.0%) [28].

Attitudes towards HH among nurses in secondary care hospital seem to be less optimal (Table 2). Around (75.0%) of the nurses disagreed with the statement “sometimes I have more important things to do than hand hygiene” compared to (80.0%) in another study in India [29]. Our findings indicate that a quarter of the nurses believe that there are important issues that justify neglecting good HH practices, which save the life of their patients. Furthermore, (74.6%) of the nurses disagreed with the statement “wearing gloves reduces the need for hand washing” compared to (73.7%) in a study on nurses in Saudi Arabia [30]. Although nurses generally accept the notion that wearing gloves is not a substitute for good HH practices, using gloves as an alternative for HH remained high despite interventions to improve HH [31]. In fact, some authors depicted wearing gloves as a contributing factor to poor CwHH and recommended interventions that alter the healthcare worker’s gloves use behavior as part of any initiative to improve CwHH [32, 33].

While (68.4%) of nursing staff were able to list “My Five Moments for Hand Hygiene” fully and appropriately, we found only (25.0%) of nursing staff were compliant with hand hygiene by direct observation. This is considerably low, but consistent with previous studies in the region. Previously, CwHH among nursing staff in secondary healthcare hospitals in Kuwait was estimated to be (33.0%) [22], while in Saudi Arabia (29.0%) [34]. Despite the fact that our study and the other two studies [22, 34] used different methodological approaches, the overall conclusion is consistent showing that the actual CwHH in the region is low. Studies in other settings reported higher observed CwHH including in Switzerland (93.6%) [35], UK (75%) [36], Turkey (62.5%) [37], Germany (52%) [38], and India (63.0%) [39].

While the CwHH by direct observation was low in all hospitals, it showed significant variation between different hospitals. This supports the notion that both institutional factors such as insufficient number of hand wash basins or the lack of the general institutional climate that encourages CwHH and individual factors (e.g. ignorance about the protocol of HH) can affect CwHH [40]. At the institutional level, key causes for compliance varies considerably, and efforts to address the causes in each hospital in a customized manner has resulted in sustainable and significant improvements in CwHH (increased from 47.5 to 81.0%) [41]. Although the variation in compliance by direct observation between different hospitals could be genuine, it could be due to the “Hawthorne effect” (i.e. nurses may modify their hand hygiene practices in response to their awareness of being observed). Although “Hawthorne effect” could have happened in all hospitals, nurses in particular hospitals, may reacted differently in addition to the fact that the head nurse may alert the nursing staff about the observation in some hospitals.

Based on four patient care scenarios, guided by WHO guidelines [42], we estimated the self-reported CwHH among nursing staff in secondary healthcare hospitals to be 69.5%. Previously, self-reported CwHH among nurses was estimated to be more than 90.0% [22]. However, the difference between our findings and the previous estimates could be due to different methodological approaches. We used scenarios taken from WHO manual for hand hygiene, while the previous study was done before the WHO guidelines on hand hygiene became commonly used. Of note, is the large difference between self-reported compliance and compliance by direct observation (Fig. 2), which had been reported in the previous study [22].

Unlike compliance by direct observation, which was anonymous, we were able to investigate factors associated with self-reported compliance. Female nurses had higher self-reported compliance in a univariable and multivariable analysis (Table 4). Previous study conducted on nursing students in Iran has reported better observed compliance among female nurses compared to male nurses [43]. In our study, nationality of nurses was also significantly associated with self-reported compliance in univariable and multivariable analysis. The correct knowledge on various aspects of HH showed significant association with self-reported compliance in univariable analysis and multivariable analysis, which suggests that effort to improve knowledge may ultimately improve hand hygiene practices among nursing staff.

This is the first study that estimated the CwHH among nurses in Kuwait using the HH direct observation tool developed by the WHO. We directly observed HH for 64 h and 40 min (194 sessions, each lasted for 20 min) in medical, surgical, pediatric, and emergency wards. Two independent observers estimated the compliance of nurses with HH in each session. However, the study has some limitations including the possibility that nurses may have changed their HH practices because of being observed (Hawthorne effect). This may have affected the compliance and thus it is possible that the CwHH maybe even lower than our estimate. In order to minimize the “Hawthorne effect”, we collected data on knowledge about HH by self-administered questionnaires only after the direct observation for HH was completed. Also, nurses were unaware of the direct observation as the observers were senior medical students, whom their presence in hospital wards usually does not attract nurses’ attention. The other limitation of our study is that we did not collect data from private healthcare services. Therefore, our findings cannot be extrapolated to nurses working in private healthcare services who may have different attitudes, knowledge, and CwHH compared to our study population. It is worth noting that the majority of healthcare services including secondary are owned by the government and the participation of private sector is modest [44]. Finally, we assessed CwHH based on ‘My Five Moments for Hand Hygiene’, which has its own limitations [45]. This includes the fact that it does not cover HH opportunities outside the patients’ zone, or for outpatients setting and looks at the healthcare setting as a uniform place [45].

## Conclusion

In conclusion, we have demonstrated that the observed CwHH among nursing staff in secondary healthcare hospitals is around (25.0%). Given the “Hawthorne effect”, observed compliance might be even lower than this estimate. Our study was conducted just before COVID-19 pandemic; thus, the findings may serve as a benchmark to investigate the impact of the pandemic on HH practices in our setting. Overall, efforts have to be made to improve HH practices among nurses in secondary healthcare hospitals in Kuwait. Interventions that have been used elsewhere and found to be effective [41, 46] may be tested in Kuwait. Also, our study demonstrated less optimal attitudes toward hand hygiene, e.g. (25.0%) of nurses thought that they sometimes have more important things to do than hand hygiene, which is striking because hand hygiene can prevent infections hence save lives. Efforts to improve the attitudes towards HH among nurses are essential to improve HH practices.

## Data Availability

The datasets analysed during the current study are available from the corresponding author on reasonable request.

## Abbreviations

HH: Hand hygiene
CwHH: Compliance with hand hygiene
HCAIs: Healthcare associated infections
ICUs: Intensive Care Units
